# Suppression of Adaptive Immune Cell Activation Does Not Alter Innate Immune Adipose Inflammation or Insulin Resistance in Obesity

**DOI:** 10.1371/journal.pone.0135842

**Published:** 2015-08-28

**Authors:** Manikandan Subramanian, Lale Ozcan, Devram Sampat Ghorpade, Anthony W. Ferrante, Ira Tabas

**Affiliations:** 1 Department of Medicine, Columbia University, New York, NY, 10032, United States of America; 2 Department of Pathology & Cell Biology, Columbia University, New York, NY, 10032, United States of America; 3 Department of Physiology & Cellular Biophysics, Columbia University, New York, NY, 10032, United States of America; University of Cambridge, UNITED KINGDOM

## Abstract

Obesity-induced inflammation in visceral adipose tissue (VAT) is a major contributor to insulin resistance and type 2 diabetes. Whereas innate immune cells, notably macrophages, contribute to visceral adipose tissue (VAT) inflammation and insulin resistance, the role of adaptive immunity is less well defined. To address this critical gap, we used a model in which endogenous activation of T cells was suppressed in obese mice by blocking MyD88-mediated maturation of CD11c^+^ antigen-presenting cells. VAT CD11c^+^ cells from *Cd11cCre* ^*+*^ *Myd88* ^*fl/fl*^ vs. control *Myd88* ^*fl/fl*^ mice were defective in activating T cells *in vitro*, and VAT T and B cell activation was markedly reduced in *Cd11cCre* ^*+*^ *Myd88* ^*fl/fl*^ obese mice. However, neither macrophage-mediated VAT inflammation nor systemic inflammation were altered in *Cd11cCre* ^*+*^ *Myd88* ^*fl/fl*^ mice, thereby enabling a focused analysis on adaptive immunity. Unexpectedly, fasting blood glucose, plasma insulin, and the glucose response to glucose and insulin were completely unaltered in *Cd11cCre* ^*+*^ *Myd88* ^*fl/fl*^ *vs*. control obese mice. Thus, CD11c^+^ cells activate VAT T and B cells in obese mice, but suppression of this process does not have a discernible effect on macrophage-mediated VAT inflammation or systemic glucose homeostasis.

## Introduction

Obesity is reaching epidemic proportions worldwide and is the major risk factor for type 2 diabetes mellitus [1]. The metabolic disturbances in obesity have been linked to chronic inflammation, particularly in visceral adipose tissue (VAT) [2]. While innate immune cells, notably adipose tissue macrophages (ATMs), have been implicated in the development of insulin resistance through their inflammatory functions [3–7], the relative role of adaptive immune cells is less well defined. T and B cells infiltrate VAT in proportion to weight gain [8, 9], and there is a clonal expansion of T cells, suggesting the existence of an antigen-specific response [10–12]. However, major gaps in this area remain, including the nature and source of the relevant antigens; the identity of the antigen-presenting cells (APCs) in VAT; and, most importantly, the functional role of T and B cell responses in VAT inflammation and glucose metabolism.

Previous studies using obese mice lacking all populations or selected subpopulations of adaptive immune cells have yielded varying results. For example, *Rag* ^*-/-*^ and *Scid* mice, which lack both T and B cells, are more insulin-resistant than WT mice when fed a high-fat diet (HFD) [11, 13], suggesting that T and B cell responses may be *protective* in obesity-associated inflammation and insulin resistance. Similarly, Th2 cells and regulatory T cells (Tregs) have been demonstrated to exert protective actions on obesity-induced insulin resistance, which in some cases was associated with suppressing ATM-mediated inflammation [10, 11]. However, other studies have suggested that activated T and B cells may exacerbate insulin resistance. For example, CD8^+^ T cell-depleted mice have decreased VAT inflammation and macrophage infiltration [14], and mice lacking Tbet, a Th1 cell transcription factor, have improved insulin sensitivity [15]. Moreover, Stat3 deletion specifically in T cells, which decreases IFN-γ producing CD4^+^ and CD8^+^ T cells, also have improved insulin sensitivity [16]. Another study showed that obese mice with MHC-II deleted in LysM^+^ cells have a partial decrease in VAT T cells and VAT ATMs, and this was associated with improved glucose homeostasis [17]. Similarly, B cell-deficient mice were demonstrated to have improved insulin sensitivity on a high-fat diet [18].

While the explanation for these varying results could be related to opposing effects of different T and B cell subsets, one also needs to consider issues related to the specific models used in these studies. For example, several of these manipulations were associated with significant changes in body weight and/or fat distribution in visceral *vs*. subcutaneous adipose tissue, which could affect metabolic endpoints [14, 15, 18]. Furthermore, complete lack of immune cell subsets results in significant systemic alterations that can secondarily impact metabolic and inflammatory processes. Thus, it would be extremely valuable to study how activated T and B cells affect VAT inflammation and metabolism using a model in which body weight, fat distribution, and other systemic effects related to obesity are not altered.

Toward this goal, we used a mouse model in which *activation* of tissue T cells, but not numbers of immune cell subsets in the peripheral blood and spleen, are suppressed via selective deletion of MyD88 in CD11c-expressing cells [19, 20]. The use of CD11c-MyD88 KO (*Cd11cCre* ^*+*^ *Myd88* ^*fl/fl*^) mice was motivated initially by the fact that MyD88 is required for the maturation of CD11c^+^ dendritic cells (DCs) and thus their ability to effectively activate both naïve and effector-memory T cells. Importantly, we show here that MyD88 deletion in CD11c^+^ adipose tissue *macrophages* also suppresses their ability to activate effector-memory T cells. This is a critical point given the predominance of CD11c^+^ macrophages in obese VAT. Indeed, we demonstrate that obese CD11c-MyD88 KO mice show a marked decrease in T and B cells and their cytokines in VAT without significant changes in VAT macrophages, ATM cytokines, or systemic inflammation. In this model of deficient activation of adaptive immunity with intact innate immunity, we found no significant improvement in systemic glucose homeostasis in obese mice.

## Materials and Methods

### Animals and diets

The following mice were purchased from The Jackson Laboratory: (a) 16-wk-old chow-fed C57BL/6J lean male mice (Stock # 000664); (b) 16-wk-old C57BL/6J DIO male mice, which were fed a HFD (5.2 kcal/gm, 60% Kcal from fat) for 10 wks (Stock # 380050); (c) *Myd88* ^*fl/fl*^ and *Cd11cCre* mice on a C57BL/6J background (stock # 008888 and 008068, respectively); and (d) OTII mice (stock # 004194). The *Myd88* ^*fl/fl*^ and *Cd11cCre* mice were bred together at specific pathogen free animal facility of Columbia University to generate *Cd11cCre* ^*+*^ *Myd88* ^*fl/fl*^ mice. Littermates without expression of Cre were used as controls whenever possible, but occasionally control mice were derived from *Myd88* ^*fl/fl*^ matings to achieve high enough n numbers for the experiments. These two groups of control mice, when directly compared with each other, yielded similar data for the immune-related and metabolic endpoints used in this study. To induce obesity in mice in our laboratory, 6-wk-old male mice were fed ab-libitum the same HFD used at The Jackson Laboratory (D12492, Research Diets Inc.). All animal protocols were approved by Institutional Animal Care and Use Committee, Columbia University, NY.

### Antibodies, primers, and quantitative real-time PCR

Antibodies against mouse CD45, CD11c, F4/80, CD3, CD4, CD8, CD62L, and CD44 were obtained from BD biosciences. Antibodies against MHC-II, CD86, CD19, B220, CD25, and FoxP3 were purchased from eBiosciences. The following primers were used in the study: *Ccr7* (5′-AACGGGCTGGTGATACTGAC-3′/5′-TAGGCCCAGAAGGGAAAGAAT); *Myd88* (5′-CACCTGTGTCTGGTCCATT-3′/5′-AGGCTGAGTGCAAACTTG-3′); *Tnfa* (5′-CATCTTCTCAAAATTCGAGTGACAA-3′/5′-TGGGAGTAGACAAGGTACAACCC-3′); *Il10* (5′-CATGGGTCTTGGGAAGAGAA-3′/5′-AACTGGCCACAGTTTTCAGG-3′); *Il12* (5′-AAGCTCTACAGCGGAAGCAC-3′/5′-ATCCTGGGGAGTTTCAGGTT-3′); *Il17* (5′-TCTCTGATGCTGTTGCTGCT-3′/5′-AGGAAGTCCTTGGCCTCAGT-3′); *Mcp1* (5′-CCCCACTCACCTGCTGCTACT-3′/5′-TTTACGGGTCAACTTGACATTC-3′); *Tgfb* (5′-GGACTCTCCACCTGCAAGAC-3′/5′-GACTGGCGAGCCTTAGTTTG-3′); *Ifng* (5′-GCGTCATTGAATCACACCTG-3′/5′-TGAGCTCATTGAATGCTTGG-3′). Primers for *Mertk*, *Cd64*, *Il6*, *Ccl17*, *Ccl19*, and *Ccl22* were purchased from Qiagen. RNA was isolated from tissues and cells using RNeasy Mini Kit (Qiagen) and was converted to cDNA using Superscript VILO cDNA synthesis kit (Invitrogen) according to the manufacturer’s protocol. Gene expression was analyzed by quantitative real-time PCR (qRT-PCR) using standard curve method on an ABI 7500 real time PCR machine.

### Stromal vascular cell fraction preparation

The mice were anaesthetized by isoflurane inhalation and blood was withdrawn by intracardiac puncture following which the mice were perfused with 10 ml of 1X PBS. Epididymal fat pads were minced in 1X Liberase in PBS and incubated for 1 h at 37°C in a shaking water bath (100 rpm). The digested tissue was strained through a 100-μm nylon mesh and resuspended in 1X PBS. The material was centrifuged at 500 x *g* for 15 min, and the pellet was collected as the stromal vascular cell fraction.

### Immunostaining, flow-cytometry, and cell sorting

Single cell suspensions obtained from SVF and spleen and peripheral blood cells obtained after ACK buffer-induced RBC lysis were incubated with Fc-block (anti-CD16/CD32) for 15 min on ice, followed by 30 min incubation with fluorophore conjugated primary antibodies as indicated in the figure legends. Following one wash with PBS, the samples were analyzed on a BD FACS CantoII or BD FACS Calibur, and the data were plotted using FlowJo. Cell sorting following immunostaining for CD11c and F4/80 was conducted on an Influx cell sorter at the CCTI flow core facility of Columbia University. Purity of the sorted population was analyzed by flow-cytometry, and we routinely recovered >95% cells that expressed the intended cell surface marker.

### Ova-OTII antigen presentation assay

Flow-sorted splenic DCs, VAT CD11c^+^F4/80^-^, and CD11c^+^F4/80^+^ cells were loaded with endotoxin-free ovalbumin (10 μg/ml, Invivogen) and co-cultured for 72 h with the following types of T cells labeled with 5 μM CFSE: CD4^+^ T cells obtained from naïve OTII mice; or effector/memory CD4^+^ T cells obtained from Ova peptide-primed OTII mice [21]. T cell proliferation was analyzed by flow-cytometry for CFSE dye dilution as described previously [22].

### Enzyme-linked immunosorbent assay (ELISA)

At the time of euthanasia, blood was collected in non-heparinized tubes via intracardiac puncture. The blood was allowed to clot, and the serum was separated by centrifugation at 10,000 x *g* for 15 min. Commercially available ELISA kits for the detection of MCP-1, TNF, IL-6, IL-1β, and IL-10 were purchased from eBiosciences, and the concentrations of these cytokines in the serum were measured following the manufacturer’s protocol.

### Measurement of metabolic parameters

After a 5-h fast, blood glucose concentration was measured using a standard glucometer and glucose test strips (One touch, Ultra), and blood was collected for quantification of plasma insulin using an ultrasensitive insulin ELISA kit (Crystal Chem). Plasma triglyceride concentration was measured using Triglyceride M Color B kit (Wako). Glucose tolerance tests were conducted on mice fasted overnight and then injected i.p. with 1 g/kg glucose. Blood glucose was measured at 15, 30, 60, 90, and 120 min post-glucose injection. Insulin tolerance tests were performed on 5 h fasted mice by measuring blood glucose at 15, 30, 60, 90, and 120 min following intraperitoneal injection of 0.75 IU/kg regular human insulin.

### Statistical analysis

All data are expressed as mean ± SEM. A two-tailed Student’s *t* test or one-way ANOVA with a Bonferroni multiple comparison posttest was used to analyze data, which in the experiments herein fit within a normal distribution. A P value of less than 0.05 was considered statistically significant.

## Results

### Visceral adipose tissue CD11c^+^F4/80^-^ cells activate naïve T cells

**Fig 1:**
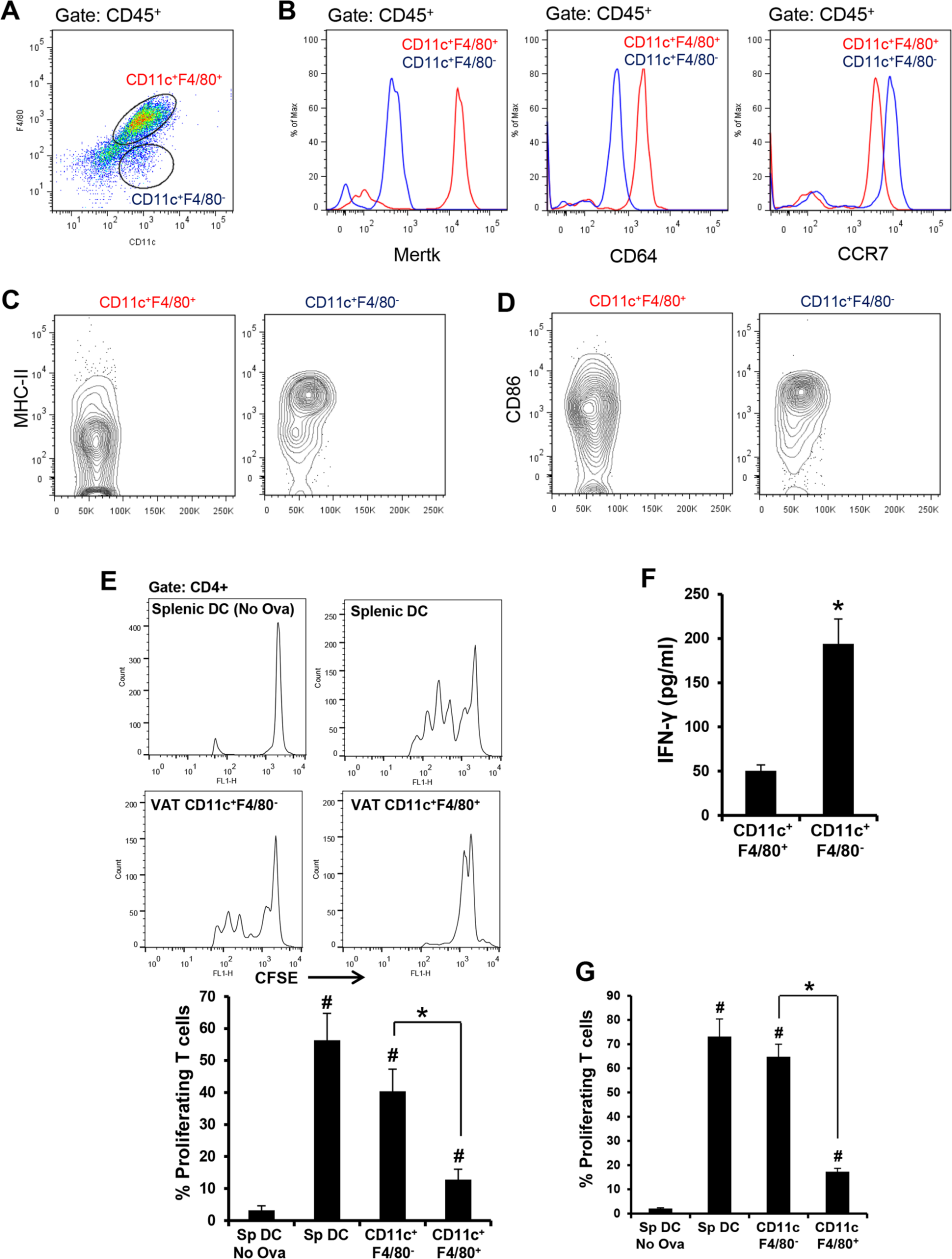
CD11c^+^ F4/80^-^ and CD11c^+^ F4/80^+^ cells from the VAT of lean and obese mice have characteristics of dendritic cells and macrophages, respectively. (A) Representative data of flow cytometric analysis of SVF cells obtained from the epididymal fat pad of 16 wk/o chow-fed male C57BL/6J mice. The dot-plot shows CD11c and F4/80 immunostaining in CD45^+^-gated SVF cells. (*B*) Representative data of flow-cytometric analysis of cell surface expression of Mertk, CD64, and CCR7 in CD11c^+^F4/80^+^ and CD11c^+^F4/80^-^ SVF cells. n = 3. (C and D) Representative data of flow cytometric analysis (contour plot) of cell-surface MHC-II and CD86 respectively in the indicated population of SVF cells. n = 3. (E) FACS-sorted splenic DCs (Sp DC) and VAT-SVF-derived CD11c^+^ F4/80^+^ and CD11c^+^ F4/80^-^ cells from lean mice were loaded with OVA and co-cultured with CFSE-labeled naïve CD4^+^ OTII transgenic T cells for 72 h. The histograms indicate proliferation of transgenic T cells as measured by dilution of CFSE dye. The bar graph represents the quantified data (*, p < 0.05 *vs*. CD11c^+^F4/80^+^ group; # p < 0.05 *vs*. Splenic DC-No Ova group). The data are representative of two independent experiments. (F) IFN-γ ELISA of cell culture supernatants of the cells used for the experiment in panel D. (G) FACS-sorted splenic DCs (Sp DC) and VAT-SVF-derived CD11c^+^ F4/80^+^ and CD11c^+^F4/80^-^ cells from DIO mice were loaded with OVA and co-cultured with CFSE-labeled naïve CD4^+^ OTII transgenic T cells for 72 h. Splenic DCs without ovalbumin served as control (*, p < 0.05 *vs*. CD11c^+^F4/80^+^ group; #, p < 0.05 *vs*. splenic DC- No Ova group). The data are representative of two independent experiments.

Before tackling the major objective of this project, we first addressed the critical issue of identifying and elucidating the functional attributes of VAT cells that are able to present antigen to and thereby activate naïve T cells [23–25]. This property characterizes DCs, which are the most efficient class of APC. We first studied APCs from the epididymal VAT of chow-fed lean mice. The stromal vascular fraction (SVF) revealed two populations of CD45^+^CD11c^+^ cells that could be distinguished based on their relative cell-surface expression of F4/80: a major population (~80%) consisting of CD11c^+^F4/80^+^ cells and a minor population (~5%) consisting of CD11c^+^F4/80^-^ cells (**Fig 1A and S1 Fig**). We found that expression of the macrophage-specific markers, MerTK and CD64 [26], was significantly higher in the CD11c^+^ F4/80^+^ cells (**Fig 1B**). Conversely, the expression of CCR7, a chemokine receptor that is critical for emigration of DCs from tissues into lymph nodes [27], was significantly higher in the CD11c^+^ F4/80^-^ cell population (**Fig 1B**). Similarly, MHC-II and the co-stimulatory molecule CD86, which enable DCs to activate naïve T cells, were also enriched in CD11c^+^ F4/80^-^ cells (**Fig 1C and 1D**).

While these data suggest that CD11c^+^ F4/80^+^ and CD11c^+^ F4/80^-^ cells may represent resident VAT macrophages and DCs, respectively, the most important functional distinction among different classes of APCs, as alluded to above, is their relative ability to present antigens to and activate naïve T cells. To investigate this property, FACS-sorted CD11c^+^ F4/80^+^ and CD11c^+^ F4/80^-^ cells were loaded with ovalbumin as a model antigen and then co-cultured with CFSE-labeled naïve CD4^+^ OTII transgenic T cells, which express a T cell receptor specific to Ova_323–339_ peptide [28] and can be assessed for proliferation by assaying CFSE dilution. Whereas VAT CD11c^+^ F4/80^-^ cells induced robust proliferation of naïve OTII T cells, which was comparable to the T cell proliferation obtained with splenic DCs, VAT CD11c^+^ F4/80^+^ cells were poor at inducing naïve T cell proliferation (**Fig 1E**). Consistent with these data, higher levels of IFN-γ were detected in the cell-culture supernatants of OTII cells incubated with CD11c^+^F4/80^-^ cells *vs*. CD11c^+^ F4/80^+^ cells (**Fig 1F**). Similar to these data with CD11c^+^ cells from lean VAT, CD11c^+^ F4/80^+^ cells from *obese* VAT had higher expression levels of *Mertk* and *Cd64* and a lower level of *Ccr7* as compared with CD11c^+^ F4/80^-^ cells from obese VAT (**S2 Fig**). Most importantly, CD11c^+^ F4/80^-^ cells from obese VAT had the unique ability to stimulate proliferation of naïve OTII T cells (**Fig 1G**). The combination of these phenotypic and functional data suggest that CD11c^+^ F4/80^+^ cells in both lean and obese VAT function as macrophages, whereas CD11c^+^F4/80^-^ cells function as DCs.

### MyD88 deficiency in VAT CD11c^+^ cells suppresses their ability to activate T cells

**Fig 2:**
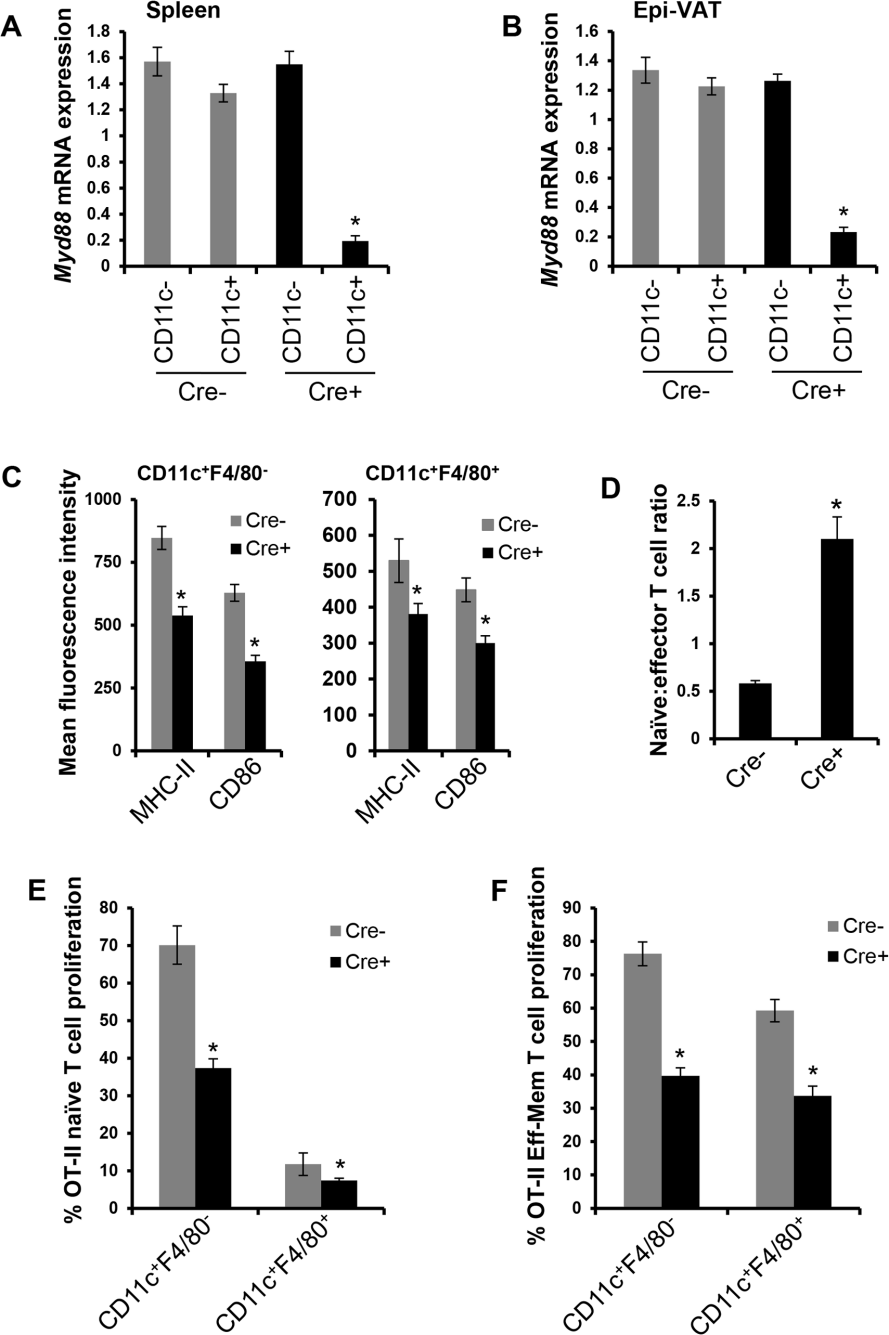
Deletion of MyD88 in VAT CD11c^+^ cells suppresses their ability to activate T cells. (A and B) *Myd88* mRNA expression was measured by qRT-PCR in FACS-sorted CD11c^-^ and CD11c^+^ cells obtained from spleens and epididymal VAT of *MyD88* ^*fl/fl*^ (Cre-) and *Cd11cCre* ^*+*^ *Myd88* ^*fl/fl*^ (Cre+) DIO mice. n = 3 mice per group (*, p < 0.05 *vs*. CD11c- group). (C) Flow-cytometric analysis of cell-surface MHC-II and CD86 in VAT CD11c^+^ F4/80^-^ (left panel) and CD11c^+^ F4/80^+^ cells (right panel) from Cre- and Cre+ DIO mice. n = 5 mice per group (*, p < 0.05 *vs*. Cre- group). (D) Flow-cytometric quantification of the ratio of naïve (CD3^+^CD62L^hi^CD44^lo^) to effector (CD3^+^CD62L^lo^CD44^hi^) T cells in the spleens of Cre- and Cre+ DIO mice. n = 5 mice per group (*, p < 0.05 *vs*. Cre- group). (E) FACS-sorted CD11c^+^ F4/80^-^ and CD11c^+^ F4/80^+^ cells from the VAT of Cre- and Cre+ DIO mice (n = 2 mice per group) were loaded with ovalbumin and co-cultured with CFSE-labeled naïve CD4^+^ OTII T cells for 72 h. T cell proliferation was measured by CFSE dye dilution by flow-cytometry (*, p < 0.05 *vs*. Cre- group). (F) Similar to (E) except that T cells that exhibited effector-memory phenotype (CD62L^lo^CD44^hi^) were obtained from Ova-primed OTII mice (*, p < 0.05 *vs*. Cre- group).

We next turned to obese CD11c-MyD88 KO mice to assess the ability of VAT CD11c^+^ DCs to activate T cells *in vitro*. This model has proven to be very valuable at addressing this type of question, because the TLR-MyD88-mediated DC maturation is required for optimal DC-mediated T cell activation [19, 20, 29]. To begin, we validated Cre-mediated recombination of floxed-*Myd88* in CD11c^+^ cells under conditions of high-fat diet (HFD) feeding: *Myd88* mRNA was decreased by ~90% in the CD11c^+^ cells of spleen and VAT of CD11c-MyD88 KO mice compared with these cells in control *Myd88* ^*fl/fl*^ mice (**Fig 2A and 2B**). Note that NK-cells and activated T cells, which express low levels of CD11c, do not show significant deletion of *Myd88* in the CD11c-MyD88 KO mice [19]. Consistent with deficient APC maturation in CD11c-MyD88 KO mice, VAT CD11c^+^F4/80^-^ and CD11c^+^F4/80^+^ cells demonstrated decreased expression of cell-surface MHC-II and CD86 (**Fig 2C**). Furthermore, the spleens of CD11c-MyD88 KO mice had an increased naïve:effector T cell ratio compared with control mice (**Fig 2D**), which is indicative of suppressed DC-mediated T cell activation *in vivo*. In *ex-vivo* experiments, we found that VAT CD11c^+^ F4/80^-^ cells from CD11c-MyD88 KO obese mice were less potent at activating naïve OTII T cells than CD11c^+^ F4/80^-^ cells from control obese mice, while CD11c^+^F4/80^+^ cells were weak at activating naïve T cell (**Fig 2E**).

Most CD11c^+^ cells in VAT have macrophage-like properties (above), and because macrophages can activate effector-memory T cells, we asked whether MyD88 in macrophages is necessary for the ability of these cells to optimally activate effector-memory T cells. Indeed, this question is fundamental to an important function of macrophages, and yet, to the best of our knowledge, the role of MyD88 in the antigen-presenting ability of macrophages has not been directly addressed in the literature. As mentioned above, we first showed that cell-surface MHC-II and CD86 were lower on CD11c^+^ F4/80^+^ cells in the Cre+ cohort. We then tested directly whether MyD88 deficiency affects the ability of CD11c^+^F4/80^+^ macrophage-like cells to activate effector-memory T cells. The data show that both types of CD11c^+^ cells activated effector-memory T cells, and in *both* cases MyD88 deficiency suppressed T cell activation (**Fig 2F**). Thus, MyD88 deficiency decreases the ability of VAT dendritic-like cells to activate both naïve T cells and effector-memory T cells and also decreases the ability of VAT macrophage-like cells to activate effector-memory T cells.

**Fig 3:**
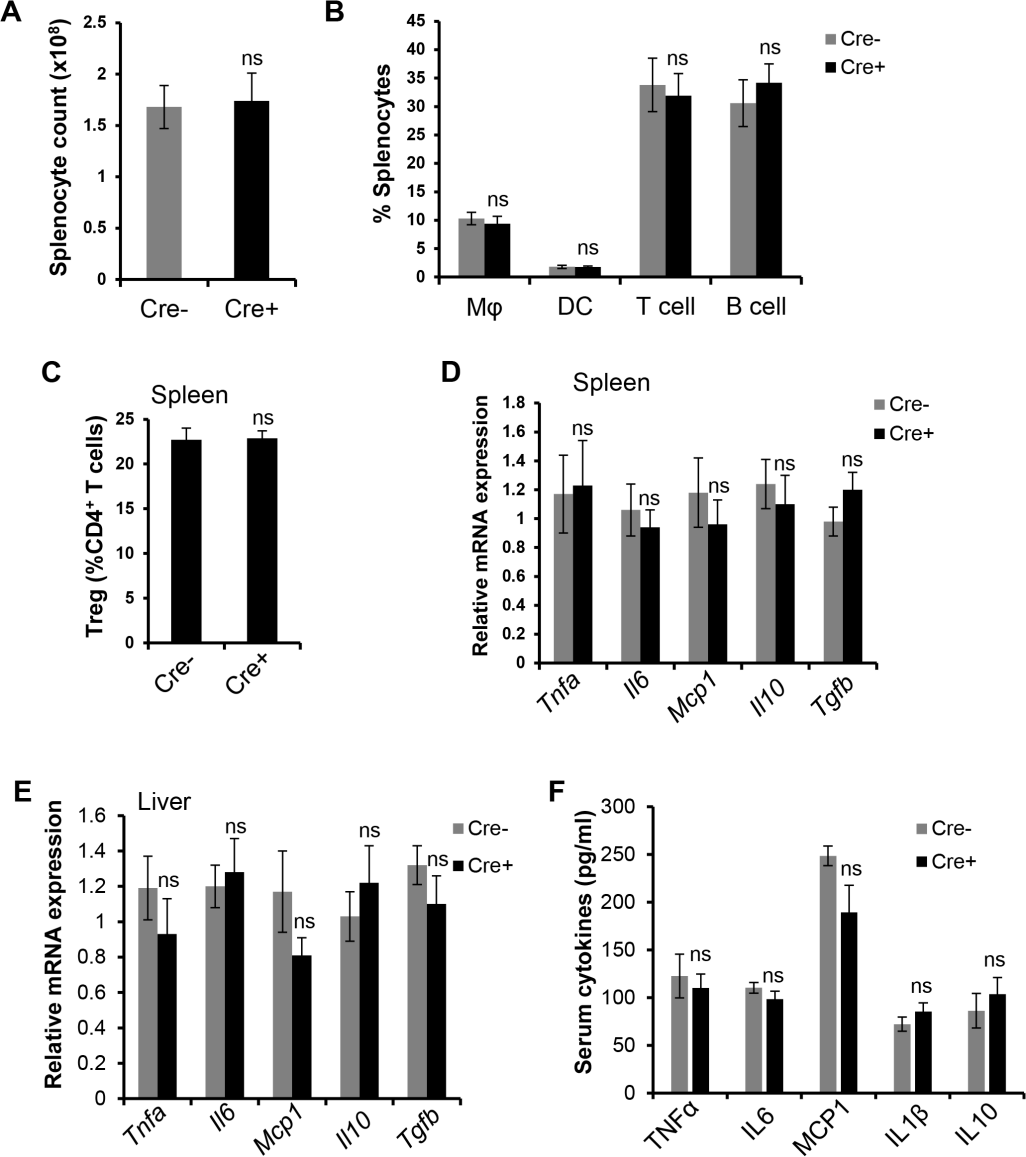
Absence of systemic immune and inflammatory changes in CD11c-MyD88 KO DIO mice. (A-C) Total splenocyte count, percent splenic macrophages (Mφ), DCs, T cells, B cells, and Tregs in Cre- and Cre+ DIO mice. n = 5 mice per group (ns, no significant difference *vs*. Cre- group). (D-E) qRT-PCR analysis of *Tnfa*, *Il6*, *Mcp1*, *Il10*, *and Tgfb* mRNA in the spleens and livers of Cre- and Cre+ DIO mice. n = 3 mice per group (ns, no significant difference *vs*. Cre- group). (F) ELISA-based measurement of TNF, IL-6, MCP-1, IL-1β, and IL-10 in serum of Cre- and Cre+ DIO mice. n = 5 mice per group (ns, no significant difference *vs*. Cre- group).

These data help establish the CD11c-MyD88 KO model as one that can be useful in addressing the consequences of defective CD11c^+^ cell-mediated T cell activation in the setting of obesity. However, if this model also affected systemic inflammation, it would not be useful to assess the role of adaptive immunity in VAT inflammation and metabolism. Previous work has shown that lean CD11c-MyD88 KO mice, despite the expected suppression of splenic T cell activation, do *not* have alterations in systemic immune parameters [20]. We now show that obese CD11c-MyD88 KO mice also do not have changes in systemic inflammation or peripheral immune cell numbers, as reflected by the following parameters that were similar in CD11c-MyD88 KO *vs*. control mice: (a) numbers of total splenocytes and splenic macrophages, DCs, T cells, B cells, and Tregs (**Fig 3A–3C and S3 Fig**); (b) mRNA levels of *Tnfa*, *Il6*, *Mcp1*, *Il10*, *and Tgfb* in the spleen and liver (**Fig 3D and 3E**); (c) serum levels of TNF, IL-6, MCP-1, Il-1β, and IL-10 (**Fig 3F**); and (d) distribution of immune cells in the peripheral blood (data not shown). Thus, obese CD11c-MyD88 KO mice are a model of defective T cell activation without measurable alterations in systemic immune cell numbers and cytokines associated with obesity.

### CD11c-MyD88 KO DIO mice have a marked decrease in T and B cells in VAT

**Fig 4:**
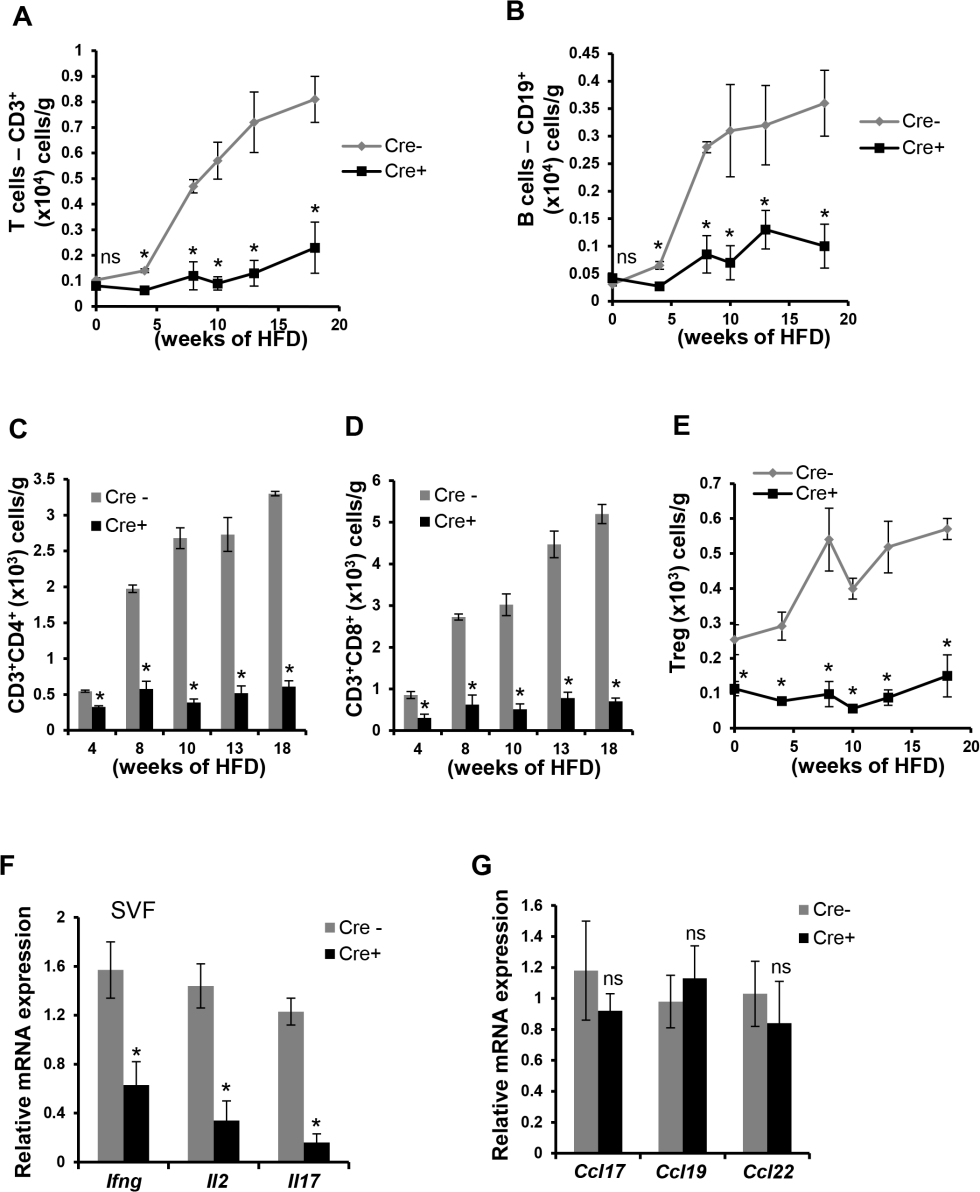
T and B cells are markedly decreased in the VAT of CD11c-MyD88 KO DIO mice. (A-E) SVF obtained from the epididymal fat pads of Cre- and Cre+ mice fed a HFD for the indicated lengths of time were analyzed by flow cytometry to quantify the numbers of total T cells (A), B cells (B), CD4^+^ T cells (C), CD8^+^ T cells (D), and Tregs (E); the data are expressed as number of cells per gram of fat (wet weight). n = 5 mice per group (*, *p* < 0.05 *vs*. Cre- group; ns, no significant difference *vs*. Cre- group). (F-G) qRT-PCR analysis of mRNA expression of *Ifng*, *Il2*, *Il17*, *Ccl17*, *Ccl19*, and *Ccl22* in SVF obtained from epididymal fat pad of 10 wk HFD-fed Cre- and Cre+ mice. n = 5 mice per group (*, *p* < 0.05 *vs*. Cre- group; ns, no significant difference *vs*. Cre- group).

Consistent with previous reports, T and B cell numbers increase in VAT after HFD feeding of WT (Cre-/-) mice (**Fig 4A and 4B, gray lines and S4 Fig**). In contrast, there was virtually no increase in T and B cells in the VAT of CD11c-MyD88 KO in response to the HFD (**Fig 4A and 4B, black lines and S4 Fig**). The lower numbers of T cells in CD11c-MyD88 KO VAT was observed across CD4^+^, CD8^+^, and Treg subclasses (**Fig 4C–4E and S4 Fig**). Moreover, there was a significant decrease in the expression of the mRNAs for the T cell-derived cytokines IFN-γ, IL2, and IL17 (**Fig 4F**). Importantly, the expression of the major T cell chemokines, *Ccl17*, *Ccl19*, and *Ccl22*, was not decreased in the VAT of obese CD11c-MyD88 KO mice (**Fig 4G**), suggesting that the decrease in VAT T cells was due to decreased T cell activation, not decreased T cell chemokinesis.

**Fig 5:**
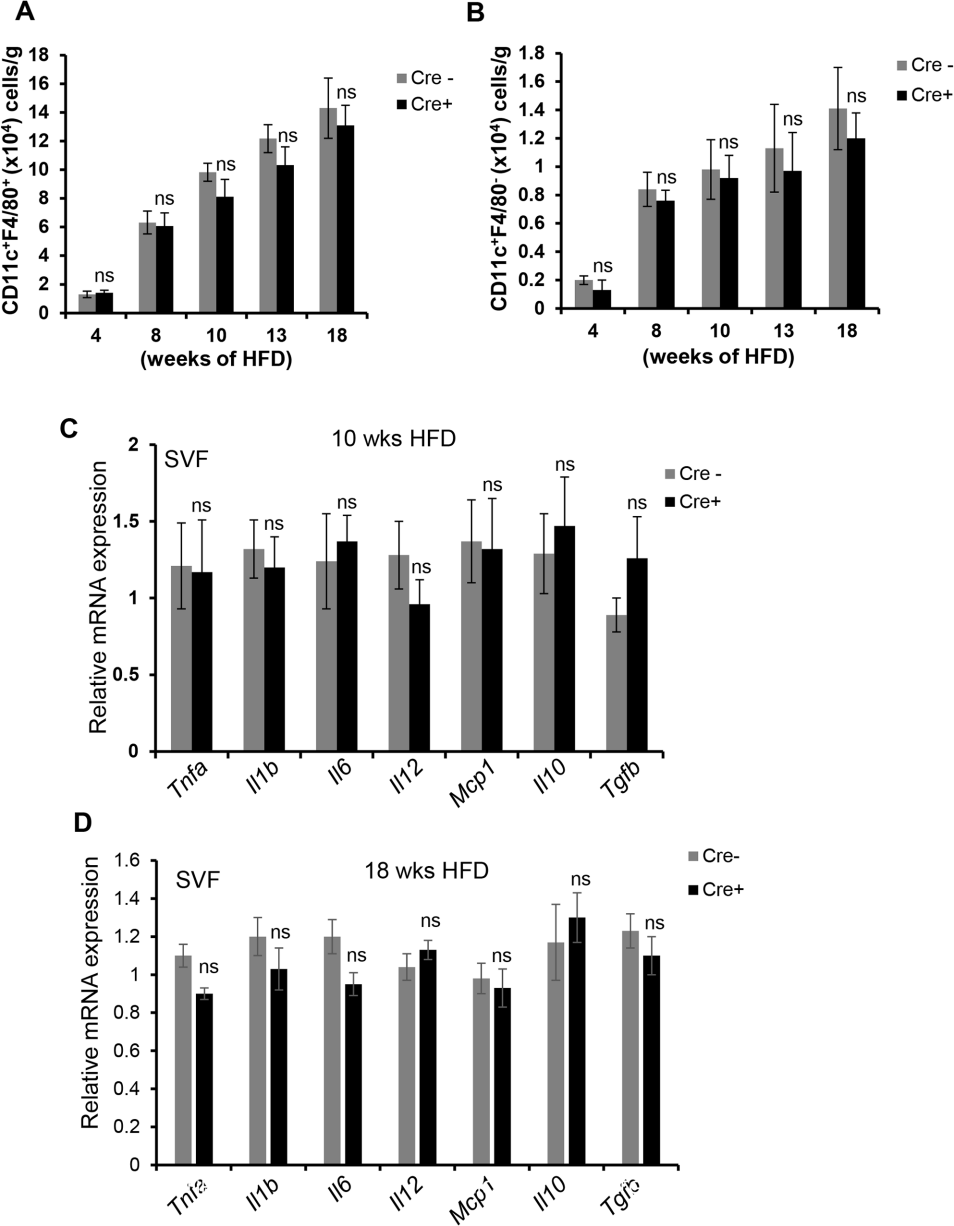
Macrophages and dendritic cell numbers and myeloid cell-derived cytokines are not altered in the VAT of CD11c-MyD88 KO DIO mice. (A-B) SVF obtained from the epididymal fat pads of Cre- and Cre+ mice fed a HFD for the indicated lengths of time were analyzed by flow cytometry to quantify the numbers of total CD11c^+^ F4/80^+^ (A) and CD11c^+^ F4/80^-^ (B) cells (n = 5 mice per group; ns, no significant difference *vs*. Cre- group). (C-D) The VAT-SVF from 10 or 18 wk HFD-fed Cre- and Cre+ mice was analyzed for expression of indicated mRNAs by qRT-PCR (n = 5 mice per group*, *p* < 0.05 *vs*. Cre- group; ns, no significant difference *vs*. Cre- group).

In striking contrast to the changes in T and B cells, the numbers of VAT CD11c^+^F4/80^+^ and CD11c^+^F4/80^-^ cells and crown-like structures were similar between the two groups of mice (**Fig 5A and 5B; S5 Fig**). Moreover, there was no difference in the expression of mRNAs for ATM-associated genes implicated in insulin resistance, including *Tnfa*, *Il1b*, *Il6*, *Il12*, and *Mcp1* (**Fig 5C and 5D**). Because the inflammatory milieu is determined by a balance between pro- and anti-inflammatory cytokines, we assayed the expression of the mRNAs for *Il10* and *Tgfb*, which again was not significantly different between the two groups of mice (**Fig 5C and 5D**). Other fat depots, including peri-renal and subcutaneous fat, also did not show differences in inflammatory gene expression between the two groups of mice (**S6 Fig**). Additionally, the decrease in T- and B-cell numbers in the VAT of obese CD11c-MyD88 KO mice was not associated with changes in either adipocyte size or lipolytic gene expression (**S7 Fig panels A and B**). Thus, despite the marked decrease in activated T and B cells in the VAT of CD11c-MyD88 KO mice, there was no significant effect on the numbers or expression of key mRNAs in VAT macrophages or adipocytes.

### The marked decrease in activated T and B cells in the VAT of CD11c-MyD88 KO DIO mice is not associated with an improvement in plasma glucose and insulin

**Fig 6:**
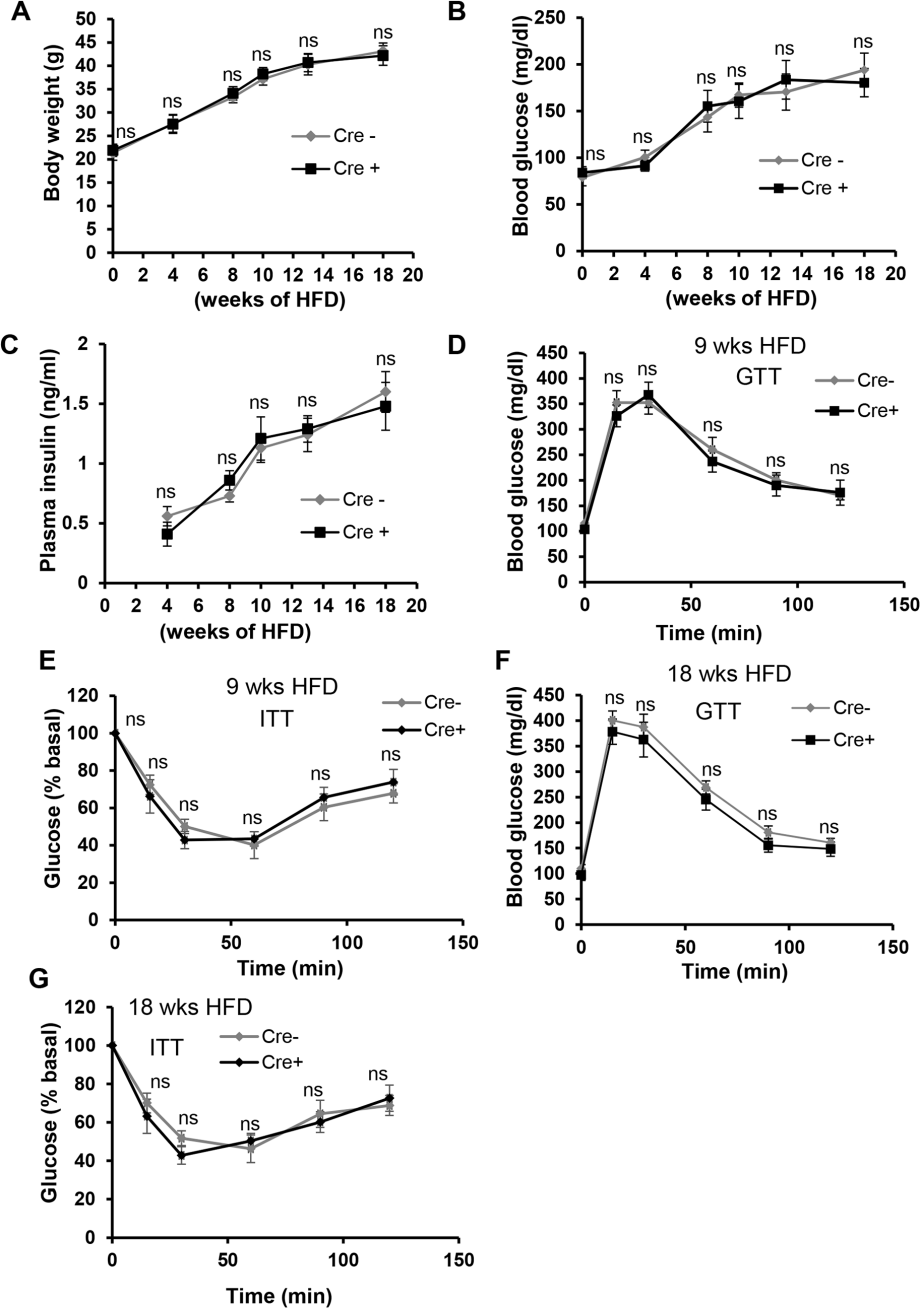
Despite the marked decrease in VAT T cells and B cells, systemic metabolic parameters are not altered in CD11c-MyD88 KO DIO Mice. (A-C) Body weight (*A*) and 5 h- fasting blood glucose (B) and plasma insulin (C) measurements in Cre- and Cre+ mice fed a high-fat diet for the indicated periods of time. n = 4–5 mice per group. (*D-E*) Glucose and insulin tolerance tests (GTT, ITT) in Cre- and Cre+ mice fed the high-fat diet for 9 wks and then fasted overnight. n = 10 mice per group (ns, no significant difference *vs*. Cre- group). (F-G) GTT and ITT in Cre- and Cre+ mice fed HFD for 18 wks and then fasted overnight. n = 5 mice per group (ns, no significant difference *vs*. Cre- group).

The data thus far have established the CD11c-MyD88 KO DIO mouse as a model to investigate the role of endogenously activated T and B cells in obesity-associated insulin resistance, because neither immune cell numbers in the periphery nor VAT myeloid-derived cells and their cytokines are affected. Both groups of mice gained weight at a similar rate and to a similar degree (**Fig 6A**). There was no difference in the weight of fat pads isolated from these mice, demonstrating no re-distribution of fat between visceral and subcutaneous depots (data not shown). Similarly, plasma triglyceride concentration was not different between the two groups of mice (215.2±18.3 vs. 207.8±11.9 mg/dl, Cre- and Cre+ mice, respectively, at 10 wks of HFD feeding). Most importantly, fasting blood glucose and plasma insulin concentrations of CD11c-MyD88 KO DIO mice were similar to those of control DIO mice (**Fig 6B and 6C**). Moreover, the blood glucose responses to glucose challenge and insulin challenge were statistically identical between the two groups of mice at 9 wks and 18 wks of HFD feeding (**Fig 6D–6G**). Thus, under conditions in which the content of T and B cells in obese VAT is markedly and selectively decreased by preventing their activation by CD11c^+^ APCs, there is no significant improvement in systemic metabolic parameters. These combined data provide support for the concept that suppression of adaptive immunity does not significantly influence VAT inflammation and VAT-mediated metabolic disturbance in obesity.

## Discussion

A number of informative studies have attempted to address the role of T and B cells in the setting of obesity and insulin resistance. However, the results of these studies have to be interpreted in the context of the models used. For example, lymphopenic mice have distinct systemic alterations that may confound the interpretation of metabolic endpoint data. Likewise, adoptive transfer of lymphocytes into lymphopenic mice, *e*.*g*., reconstitution of *Rag* ^*-/-*^ mice with CD4^+^ or CD8^+^ T cells, results in the homeostatic proliferation of the specific cells that were transferred, which might change their phenotypic and functional characteristics [30]. Other studies have blocked T cell activation by deleting MHC-II in all cells [31] or specifically in LysM^+^ cells (monocytes and monocyte-derived cells) [17]. Both of these manipulations led to improvement in insulin sensitivity. However, mice lacking MHC-II have a significant decrease in CD4^+^ T cells in the periphery that could confound the interpretation of metabolic data. Interestingly, macrophage-specific deletion of MHC-II in LysM^+^ cells led to a decrease in the number of CD11c^+^ ATMs, which by itself is known to improve insulin sensitivity [7]. It is possible that the decrease in CD11c^+^ ATMs was secondary to the decrease in T cells in this model, but a much more robust decrease in T cells in the model reported in this study did not lead to a decrease in ATMs. In view of the roles for MHC-II in enhancing monocyte migration to chemotactic signals [32] and in activation of pro-survival Btk signaling [33–35], it is possible that the decrease in ATMs was a direct effect of deletion of MHC-II in myeloid cells, *i*.*e*., by blocking monocyte migration to VAT or increasing ATM death. Other studies have used acute injection of neutralizing antibodies against T and B cells to avoid some of the above issues [12, 24, 36]. However, the short duration of this intervention, usually 1–3 weeks, precludes an evaluation of the long-term and lasting effects of the intervention, which is a critical issue when studying metabolic endpoints in chronic obesity.

In an attempt to avoid some of the shortcomings in these models, we adopted an approach in which we blocked the endogenous activation of T and B cells by suppressing the ability of CD11c^+^ APCs to activate T cells. As designed, there was a marked inhibition of T and B cell activation systemically and in the VAT of CD11c-MyD88 KO obese mice. The distinguishing feature of this model, critical for addressing the question at hand, was the absence of an effect on innate and adaptive immune cell numbers in the periphery. Also, control and experimental animals gained similar weight, showed comparable fat distribution when placed on a high-fat diet, and had similar numbers of myeloid-derived cells and their cytokines in VAT. These advantages of the current model in the context of the data presented here, suggest that activation of adaptive immune cells does not influence VAT inflammation or insulin sensitivity under conditions of obesity. A possible explanation of our findings could be related to the concomitant decrease of both pro-inflammatory effector T cells and anti-inflammatory regulatory T cells. However, *Rag* ^*-/-*^ and *Scid* mice also lack both effector and regulatory T cells, and yet in these model insulin resistance is actually worsened [11, 13]. Moreover, although VAT-Tregs have been reported to regulate the inflammatory tone of the lean adipose tissue and control insulin sensitivity [10], we found that lean CD11c-MyD88 KO mice, which have significantly lower VAT Tregs but normal levels of total T cells, showed similar glucose tolerance and insulin sensitivity as control mice. A recent study demonstrated that IL-33 can selectively expand Tregs in VAT and improve glucose tolerance in obese animals [37]. However, given that insulin signaling in VAT was not improved in this model despite restoration of VAT-Tregs, IL-33 presumably affects whole body glucose metabolism by other mechanisms. For example, IL-33 can promote the development of Th2 cells [38] and myeloid-derived suppressor cells (MDSCs) [39], both of which are reported to improve glucose tolerance in obese animals [11, 40]. In the end, the only way to address whether the decrease in Tregs in the model used here counterbalances the decrease in effector T cells to explain the null result would be to selectively and *chronically* restore Tregs in obese VAT without inducing systemic changes, and to the best of our knowledge this goal is not yet achievable.

MyD88 is a key adaptor protein for several TLR- and IL1-family receptors and is integral for activation of pro-inflammatory NF-κB signaling downstream of TLR activation [41]. In this context, it is important to note that deficiency of MyD88 has varied effects on insulin resistance depending on cell type in which it is deleted. For example, mice that have a specific deletion of MyD88 in neuronal cells are protected from HFD-induced weight gain and insulin resistance [42]. In contrast, global MyD88-deficient mice demonstrate *worse* insulin resistance upon HFD-feeding [43]. A surprising and interesting aspect of the data herein pertain to the absence of changes in VAT inflammation despite the lack of MyD88 in CD11c-expressing cells, which are the major inflammatory cells in VAT [7, 44]. In contrast to our findings, a recent study demonstrated that deletion of MyD88 in CD11b^+^ cells is associated with decreased VAT inflammation and improved insulin sensitivity [45]. Deletion of MyD88 in CD11b^+^ cells was associated with a decrease in systemic inflammation [45], which could have secondarily influenced metabolic parameters. In this regard, it is important to note that CD11b is expressed on a variety of myeloid cells, including Kupffer cells, a subset of DCs, and neutrophils, while CD11c is expressed predominantly on DCs and a subset of inflammatory macrophages.

An interesting finding from our study is that deletion of MyD88 in CD11c^+^ cells does not affect VAT inflammation aside from the expected decrease in T cell cytokines, which suggests that CD11c^+^ cell-mediated VAT inflammation in obesity is MyD88-independent. Consistent with this conclusion, we found that the production of TNFα and IL-1β and phosphorylation of p38-MAPK and NF-κB p-65 in VAT macrophages in CD11c-MyD88 KO mice were similar to that in WT obese mice, and these endpoints were also similar in cultured macrophages from MyD88-deficient and WT mice treated with palmitate (M. Subramanian and I. Tabas, unpublished data). Two previous studies also suggest the possibility that MyD88-independent signaling may be important in VAT inflammation. First, mice deficient in the two TLRs that use TRIF—TLR4 and TLR3 [46]—are protected from obesity-induced VAT inflammation and insulin resistance [47, 48]. Second, exosome-like vesicles derived from obese adipose tissue activate macrophages *in vitro* and induce the secretion of pro-inflammatory cytokines via a TLR4/TRIF pathway [49]. Thus, pending additional studies directly addressing the role of TRIF in *vivo*, VAT inflammation may be predominantly driven by TRIF-mediated signaling.

In summary, we have used a model with which we could determine whether APC-mediated T cell activation affects VAT inflammation and metabolic disturbance in the setting of obesity. The data show that MyD88 in VAT cells with dendritic- or macrophage-like properties is crucial for T cell activation *ex vivo* and for the dramatic rise in T and B cells in VAT in the setting of obesity. Most importantly, we have demonstrated a clear disconnect between VAT T and B cell activation and systemic glucose metabolism in DIO mice. These findings lend support to the concept that direct activation of innate immunity rather than adaptive immunity links VAT inflammation with metabolic disturbance in obesity, and raise the very interesting question as to what other roles VAT T and B cells may play in this setting.

## Supporting Information

S1 Fig Gating strategy for flow-cytometric analysis of SVF cells. An FSC/SSC gate was applied to eliminate debris from analysis (A) followed by exclusion of dead cells by Aqua Live/Dead staining (B). Expression of CD45 was analyzed on live cells (C). The CD45+ cell gate was used to analyzed immune cell composition in SVF (D).
(TIF)

S2 Fig Gene signatures of CD11c^+^ F4/80^+^ and CD11c^+^ F4/80^-^ cells from lean and obese VAT. RT-qPCR analysis of expression levels of *Mertk*, *Cd64*, and *Ccr7* in FACS-sorted CD11c^+^ F4/80^+^ and CD11c^+^ F4/80^-^ cells obtained from the epididymal fat pad of lean (A) or DIO (B) mice (n = 3 mice per group; *, p < 0.05 *vs*. CD11c^+^F4/80^+^ group).
(TIF)

S3 Fig Analysis of immune cell composition of splenocytes from Cre- and Cre+ fed the HFD for 10 wks. Representative flow cytometry dot-plots of splenocytes stained for F4/80 (macrophage), CD11c (DC), CD3 (T cell), CD19 (B cell), and CD4/FoxP3 (Tregs). Similar data were obtained from mice that were fed the HFD for 4, 8, 13, and 18 wks (n = 4–5 mice per group).
(TIF)

S4 Fig Analysis of immune cell composition of CD45^+^-gated SVF cells from Cre- and Cre+ mice fed the HFD for 10 wks. Representative flow cytometry contour plots demonstrating CD3, CD4, CD8, and FoxP3 staining of CD45^+^-gated SVF cells (n = 4–5 mice per group). Similar data were obtained with mice that were fed HFD for 4, 8, 13, and 18 wks.
(TIF)

S5 Fig Analysis of crown-like structures (CLS) in epididymal VAT of obese Cre- and Cre+ mice. VAT sections from 10-wk HFD-fed Cre- and Cre+ mice were immunostained with anti-F4/80 antibody, and the percentage of CLS macrophages per total cells in each section was quantified by microscopic analysis (n = 5 mice per group; ns, no significant difference).
(TIF)

S6 Fig Inflammatory gene expression in peri-renal (A) and subcutaneous (B) fat pads is similar in Cre- and Cre+ 10-wk HFD-fed mice. The mRNA of the indicated genes were assayed by RT-qPCR (n = 4–5 mice per group; ns, no significant difference).
(TIF)

S7 Fig VAT adipocyte parameters are similar in Cre- and Cre+ 10-wk HFD-fed mice. (A) Epididymal VAT sections were imaged using a 40X objective, and the mean adipocyte area was quantified using ImagePro Plus. A total of 200 adipocytes were counted per mouse. (B) The mRNA of lipoprotein lipase (*Lpl*) and adipose tissue triglyceride lipase (*Pnpla2*) was assayed by RT-qPCR. For all data, n = 5 mice per group; ns, no significant difference.
(TIF)
