# Supplementary figures and images for: Suppression of Adaptive Immune Cell Activation Does Not Alter Innate Immune Adipose Inflammation or Insulin Resistance in Obesity
Source: PLoS One. 2015 Aug 28;10(8):e0135842. doi: 10.1371/journal.pone.0135842 (PMC4552860; doi:10.1371/journal.pone.0135842)

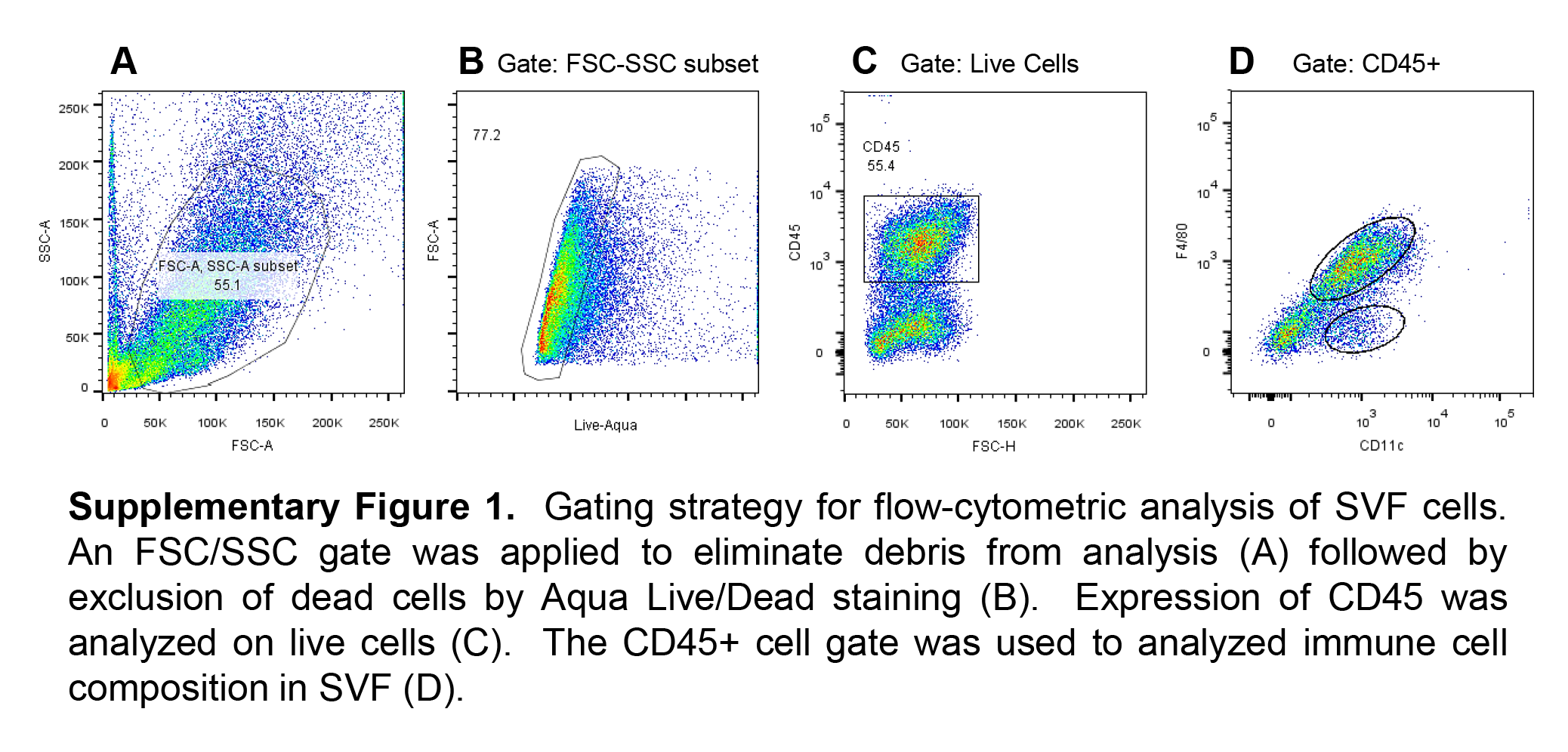

Supplement: S1 Fig — An FSC/SSC gate was applied to eliminate debris from analysis (A) followed by exclusion of dead cells by Aqua Live/Dead staining (B). Expression of CD45 was analyzed on live cells (C). The CD45+ cell gate was used to analyzed immune cell composition in SVF (D). (TIF) [file pone.0135842.s001.tif]

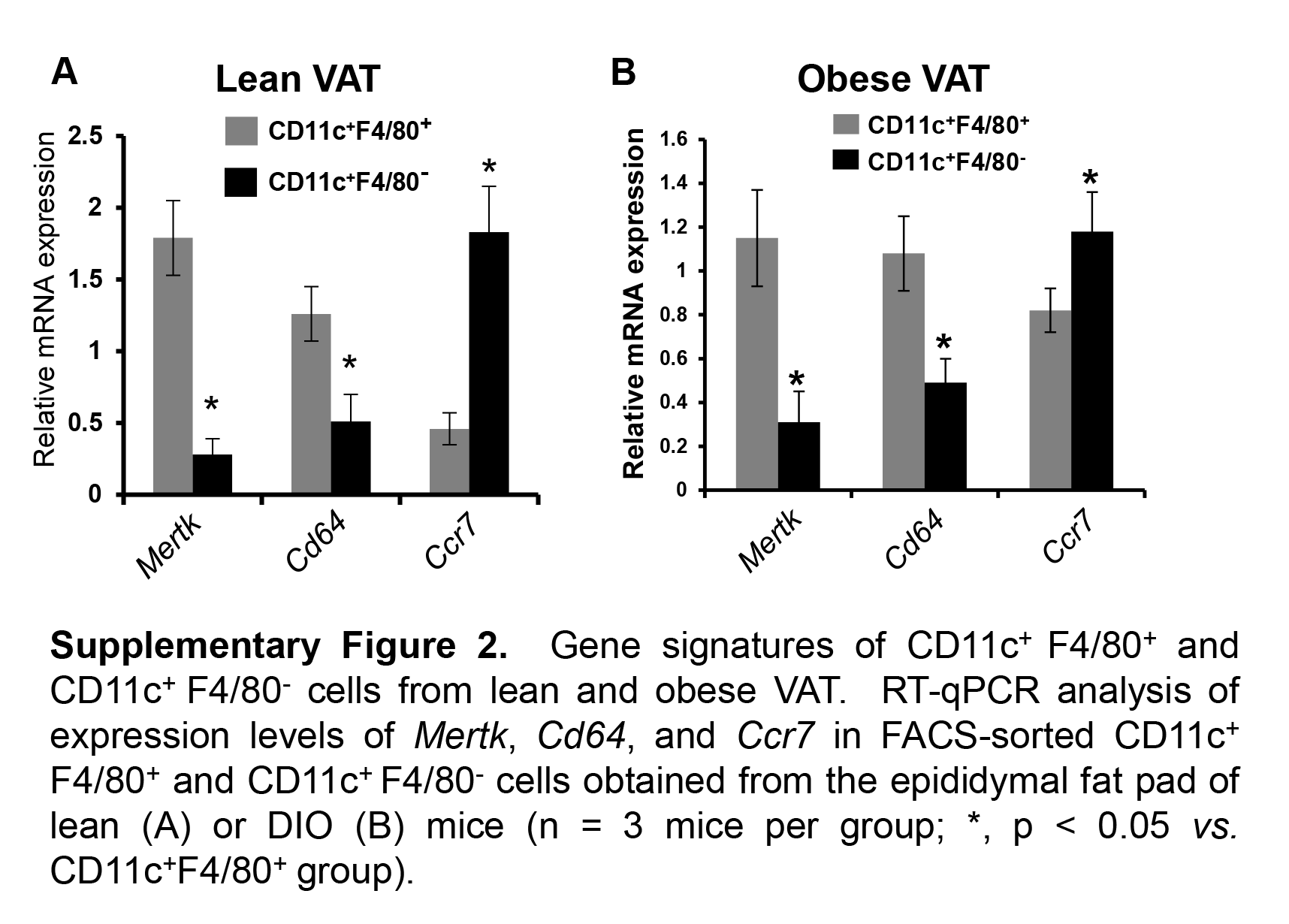

Supplement: S2 Fig — RT-qPCR analysis of expression levels of Mertk, Cd64, and Ccr7 in FACS-sorted CD11c+ F4/80+ and CD11c+ F4/80- cells obtained from the epididymal fat pad of lean (A) or DIO (B) mice (n = 3 mice per group; *, p < 0.05 vs. CD11c+F4/80+ group). (TIF) [file pone.0135842.s002.tif]

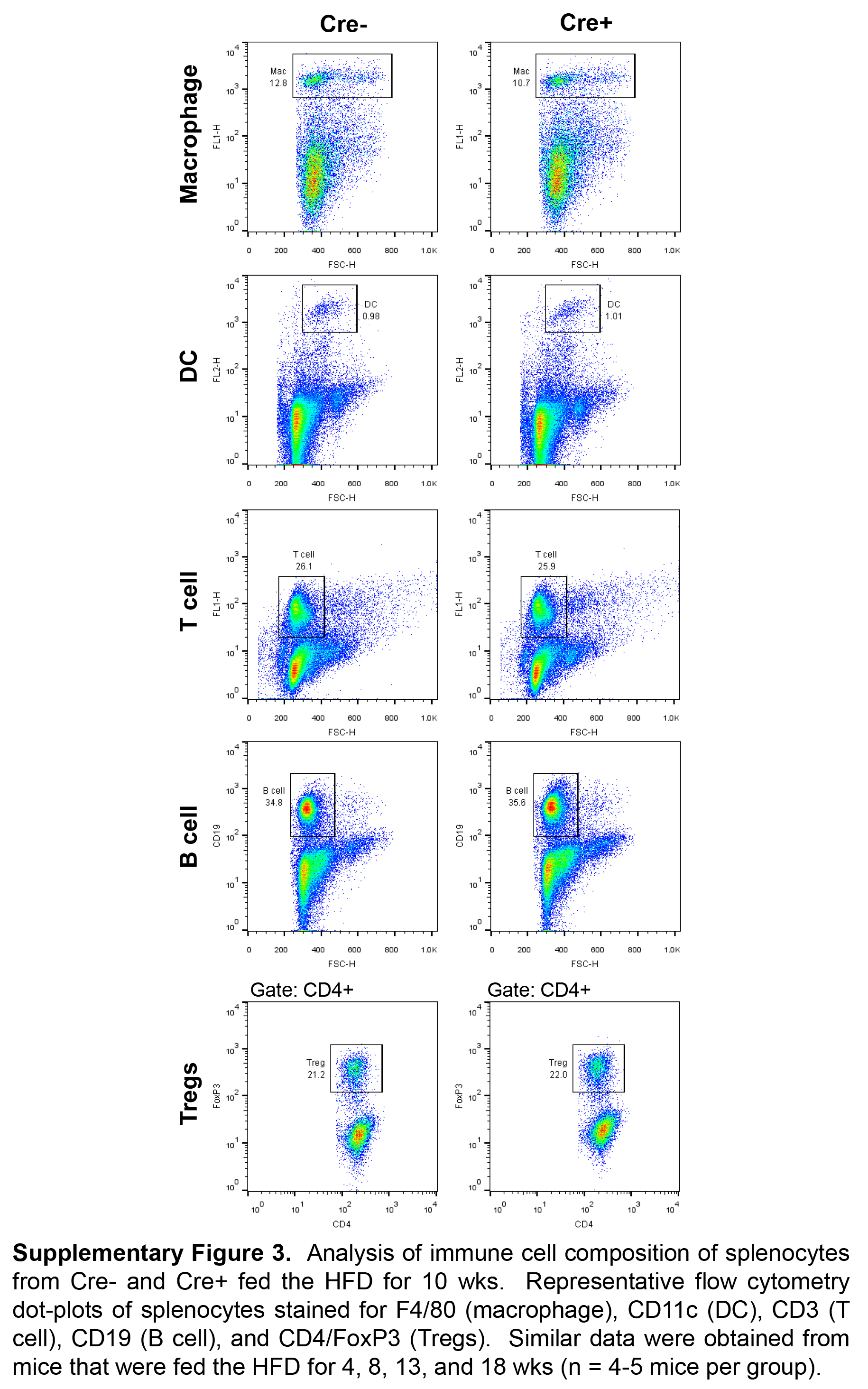

Supplement: S3 Fig — Representative flow cytometry dot-plots of splenocytes stained for F4/80 (macrophage), CD11c (DC), CD3 (T cell), CD19 (B cell), and CD4/FoxP3 (Tregs). Similar data were obtained from mice that were fed the HFD for 4, 8, 13, and 18 wks (n = 4–5 mice per group). (TIF) [file pone.0135842.s003.tif]

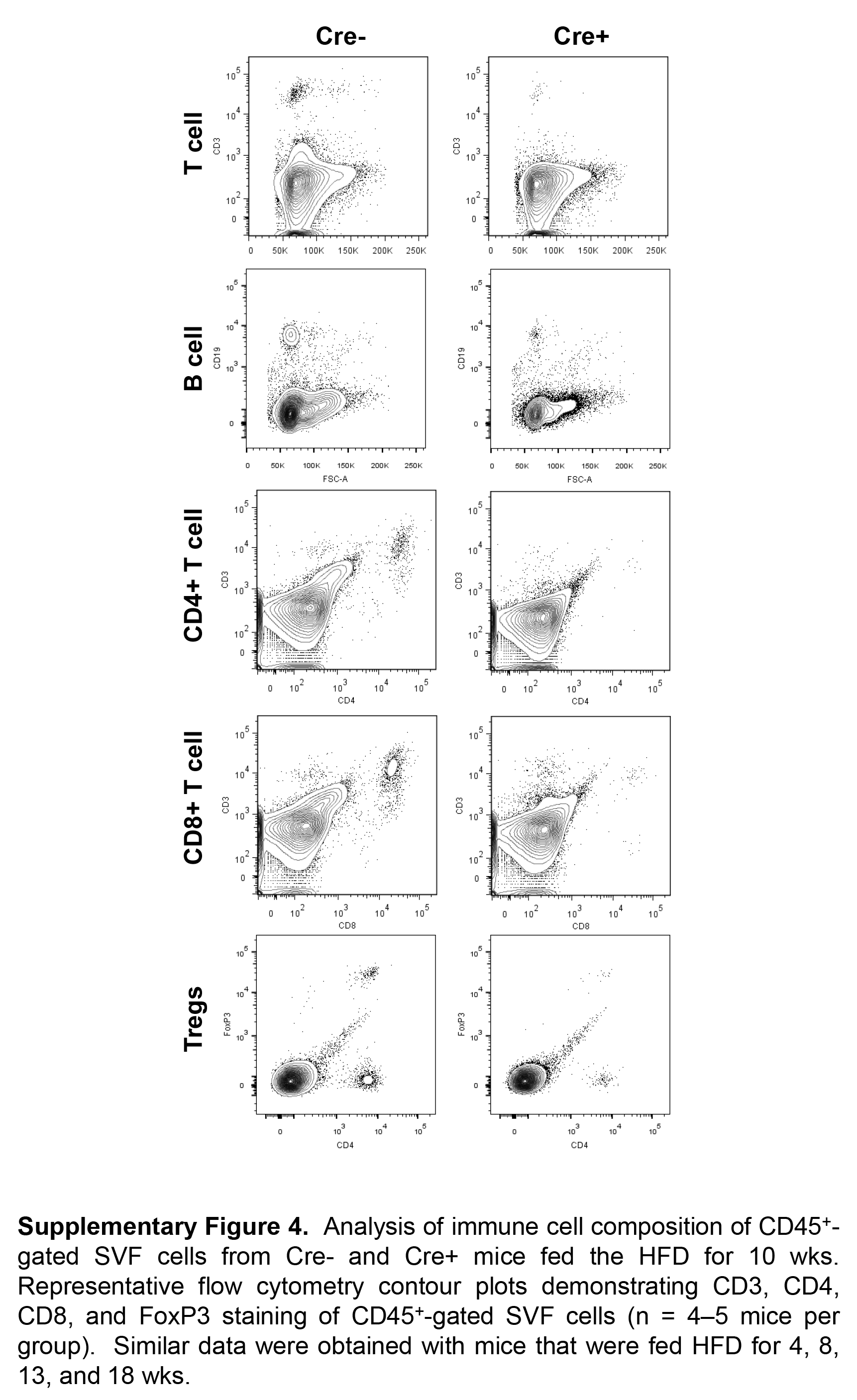

Supplement: S4 Fig — Representative flow cytometry contour plots demonstrating CD3, CD4, CD8, and FoxP3 staining of CD45+-gated SVF cells (n = 4–5 mice per group). Similar data were obtained with mice that were fed HFD for 4, 8, 13, and 18 wks. (TIF) [file pone.0135842.s004.tif]

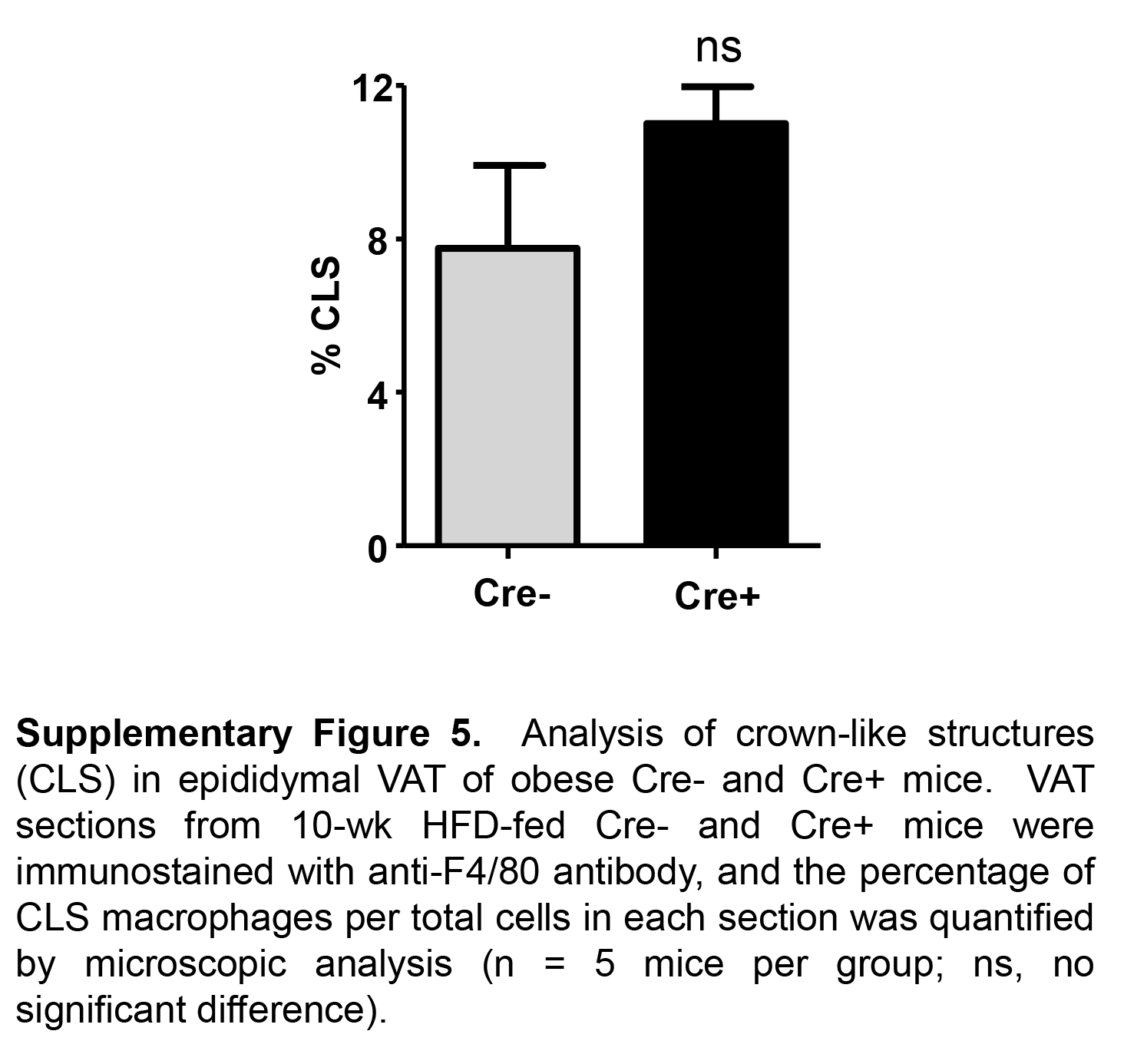

Supplement: S5 Fig — VAT sections from 10-wk HFD-fed Cre- and Cre+ mice were immunostained with anti-F4/80 antibody, and the percentage of CLS macrophages per total cells in each section was quantified by microscopic analysis (n = 5 mice per group; ns, no significant difference). (TIF) [file pone.0135842.s005.tif]

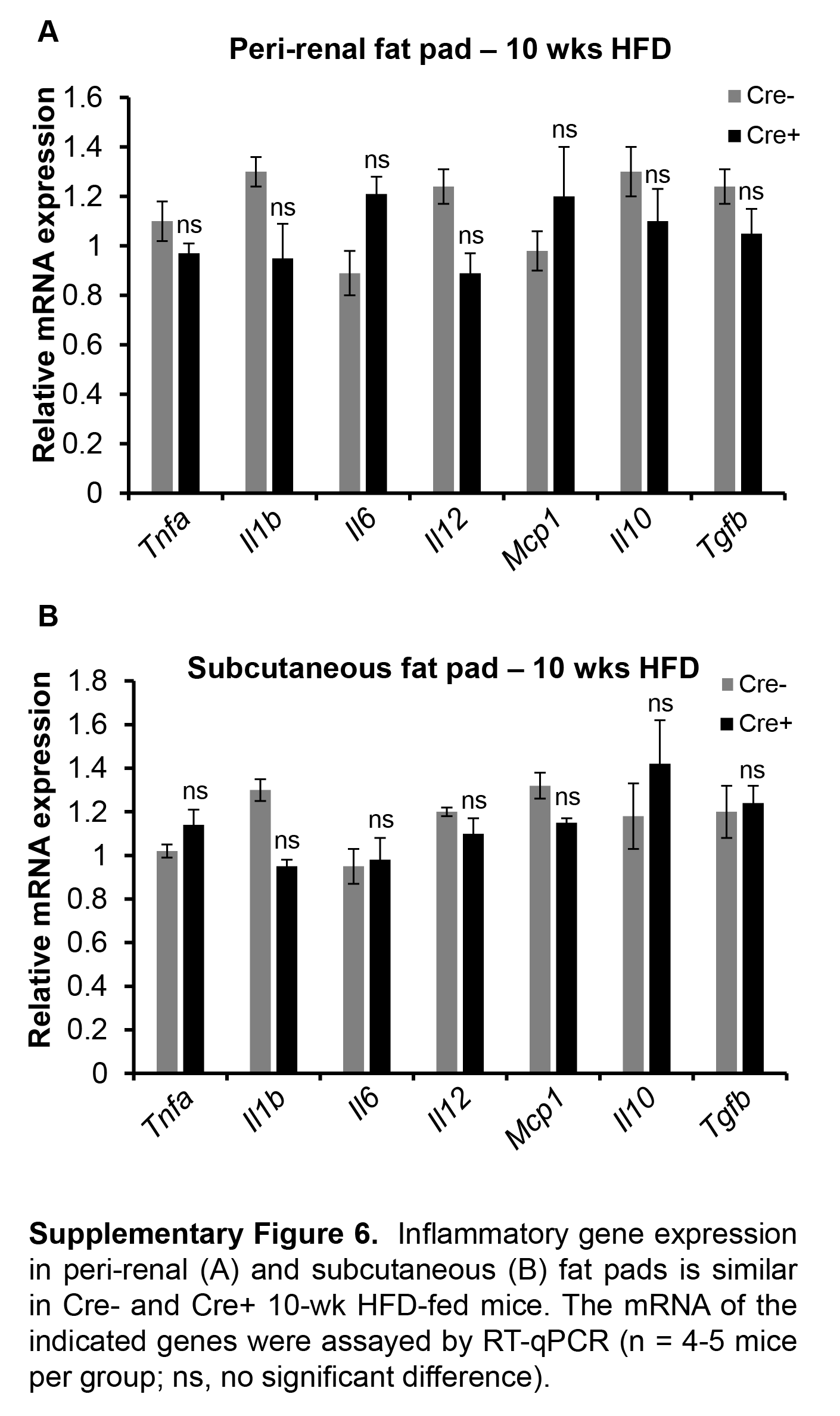

Supplement: S6 Fig — The mRNA of the indicated genes were assayed by RT-qPCR (n = 4–5 mice per group; ns, no significant difference). (TIF) [file pone.0135842.s006.tif]

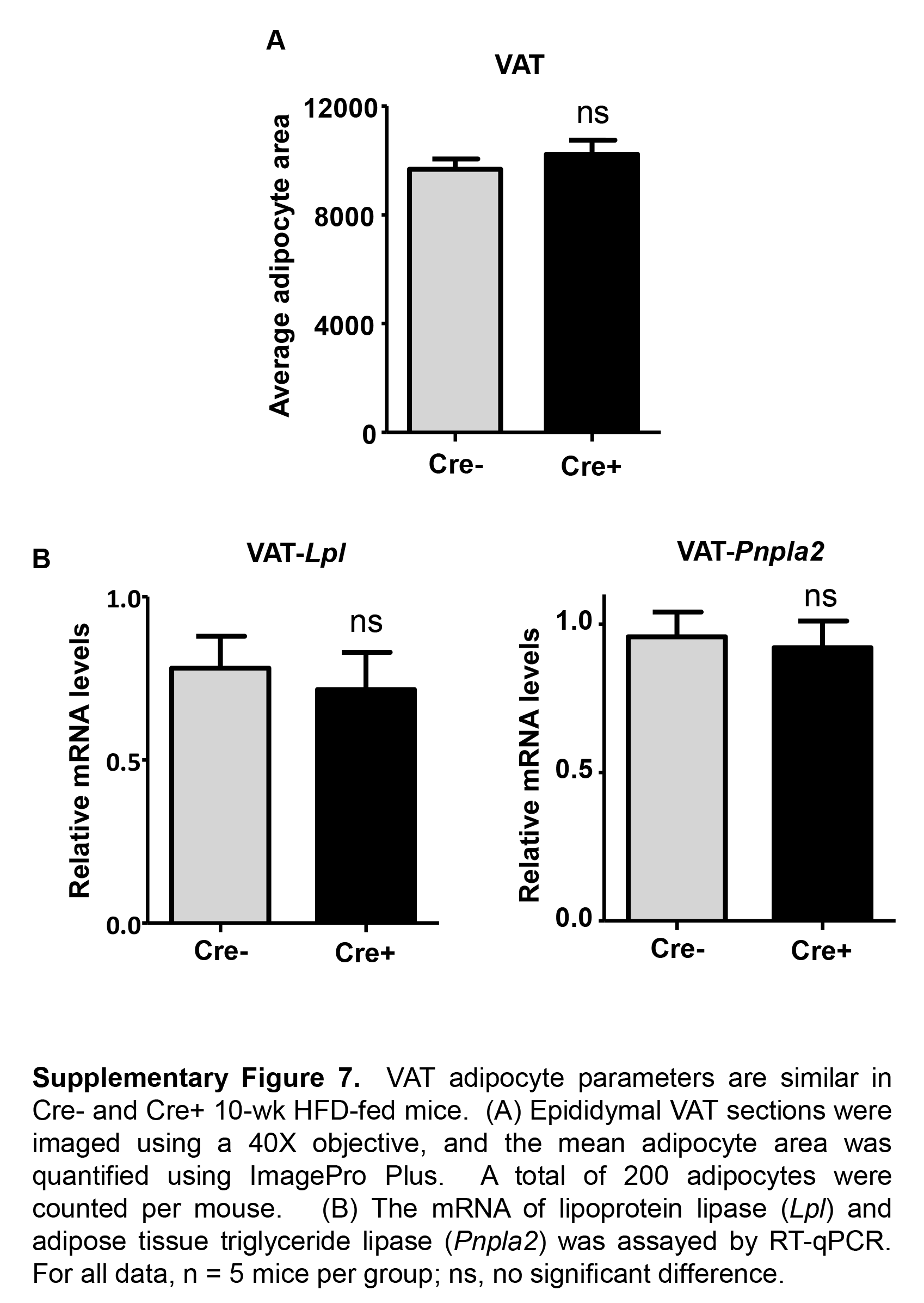

Supplement: S7 Fig — (A) Epididymal VAT sections were imaged using a 40X objective, and the mean adipocyte area was quantified using ImagePro Plus. A total of 200 adipocytes were counted per mouse. (B) The mRNA of lipoprotein lipase (Lpl) and adipose tissue triglyceride lipase (Pnpla2) was assayed by RT-qPCR. For all data, n = 5 mice per group; ns, no significant difference. (TIF) [file pone.0135842.s007.tif]
